# Supplementary material for: Basal Forebrain-Dorsal Hippocampus Cholinergic Circuit Regulates Olfactory Associative Learning
Source: Int J Mol Sci. 2022 Jul 30;23(15):8472. doi: 10.3390/ijms23158472 (PMC9368792; doi:10.3390/ijms23158472)
Supplement: Supplementary file 1 [file ijms-23-08472-s001.zip › ijms-1821159-supplementary.pdf]

International Journal of *Molecular Sciences*

MDPI

Supplementary Material

Basal Forebrain-Dorsal Hippocampus Cholinergic Circuit Regulates Olfactory Associative Learning

Yingwei Zheng [1,2,†], Sijue Tao [2,†], Yue Liu [2], Jingjing Liu [3,4], Limei Sun [1], Yawen Zheng [1], Yu Tian [2], Peng Su [3], Xutao Zhu[3,*], Fuqiang Xu [2,3,*]

[1] Jiangsu Key Laboratory of Brain Disease and Bioinformation, Research Center for Biochemistry and Molecular Biology, Xuzhou Medical University, Xuzhou 221004, China
[2] State Key Laboratory of Magnetic Resonance and Atomic and Molecular Physics, Key Laboratory of Magnetic Resonance in Biological Systems, Wuhan Center for Magnetic Resonance, Innovation Academy for Precision Measurement Science and Technology, Chinese Academy of Sciences, Wuhan 430071, China
[3] Shenzhen Key Laboratory of Viral Vectors for Biomedicine, Key Laboratory of Quality Control Technology for Virus-Based Therapeutics, Guangdong Provincial Medical Products Administration, NMPA Key Laboratory for Research and Evaluation of Viral Vector Technology in Cell and Gene Therapy Medicinal Products, The Brain Cognition and Brain Disease Institute (BCBDI), Shenzhen Institute of Advanced Technology, Chinese Academy of Sciences, Shenzhen-Hong Kong Institute of Brain Science-Shenzhen Fundamental Research Institutions, Shenzhen 518055, China
[4] University of the Chinese Academy of Sciences, Beijing 100049, China
* Correspondence: xt.zhu@siat.ac.cn (X.Z.); fuqiang.xu@wipm.ac.cn (F.X.)
† These authors contributed equally.

Citation: Zheng, Y.; Tao, S.; Liu, Y.; Liu, J.; Sun, L.; Zheng, Y.; Tian, Y.; Su, P.; Zhu, X.; Xu, F. Basal Forebrain-Dorsal Hippocampus Cholinergic Circuit Regulates Olfactory Associative Learning. *Int. J. Mol. Sci.* **2022**, *23*, 8472. https://doi.org/10.3390/ijms23158472

Academic Editor: Diego Guidolin

Received: 1 July 2022
Accepted: 28 July 2022
Published: 30 July 2022

Publisher's Note: MDPI stays neutral with regard to jurisdictional claims in published maps and institutional affiliations.

Copyright: © 2022 by the authors. Licensee MDPI, Basel, Switzerland. This article is an open access article distributed under the terms and conditions of the Creative Commons Attribution (CC BY) license (https://creativecommons.org/licenses/by/4.0/).

Supplementary Materials: The following supporting information can be downloaded at: https://www.mdpi.com/article/10.3390/ijms23158472/s1, **Figure S1**: Proportion of hippocampal outputs from cholinergic neurons in the different subpopulations of basal forebrain; **Figure S2**. The proportion of cholinergic neurons labeled by RV in three subregions of the basal forebrain; **Figure S3.** Ca^{2+} signal of mice cholinergic neurons changed in control and GCaMP6 groups during lick-ing behavior; **Figure S4.** Ca^{2+} signal of mice cholinergic neurons changed in control groups in the initial and final stage of go/no-go learning; Appendix Table: Abbreviations.

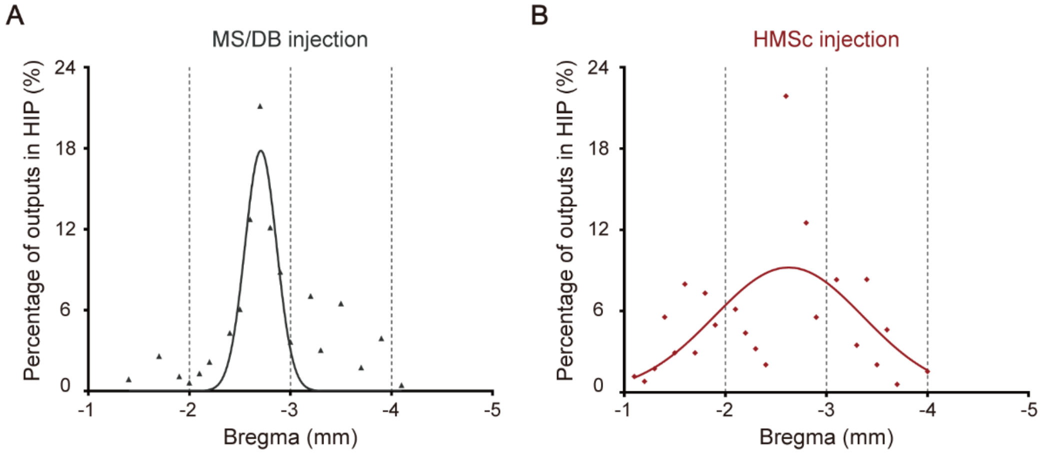

Supplementary Figure S1. Proportion of hippocampal outputs from cholinergic neurons in the different subpopulations of the basal forebrain.

(A) The anterior-posterior distribution of hippocampal neurons labeled with HSV in the MS/DB injection group. (B) The anterior-posterior distribution of hippocampal neurons labeled with HSV in the HMSc injection group. The percentage of signals in each coronal slice was calculated and fitted to a Gaussian curve.

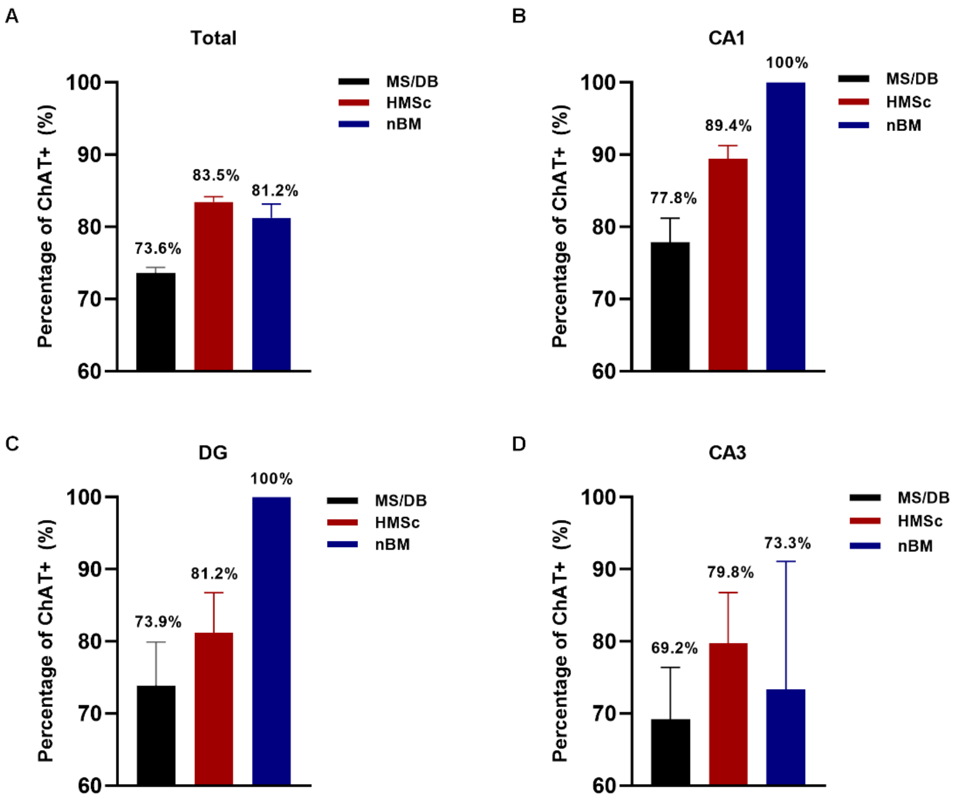

Supplementary Figure S2. The proportion of cholinergic neurons labeled by RV in three subregions of the basal forebrain.

(A) Percent of ChAT+ neurons labeled by RV in three subregions of the basal forebrain. (B) Percent of cholinergic neurons projecting to CA1 in three subregions of the basal forebrain. (C) Percent of cholinergic neurons projecting to DG in three subregions of the basal forebrain. (D) Percent of cholinergic neurons projecting to CA3 in three subregions of the basal forebrain. Three subregions of the basal forebrain included MSDB, HMSc and nBm.

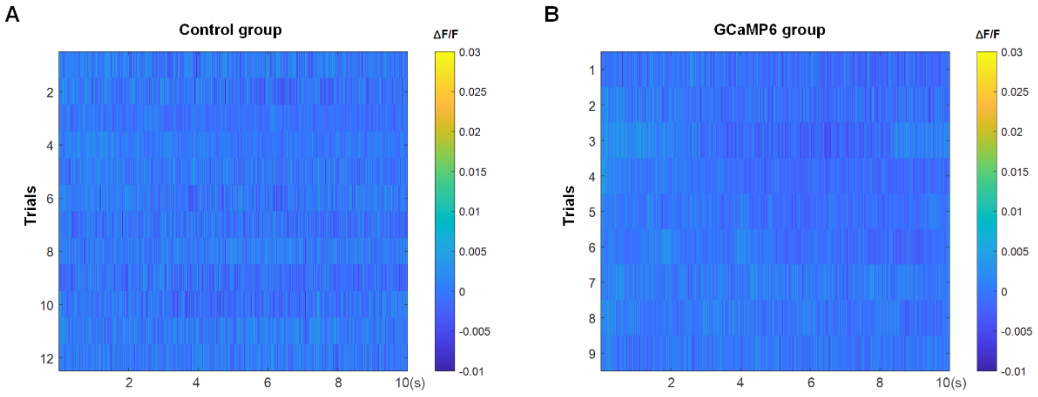

Supplementary Figure S3. Ca^{2+} signal of mice cholinergic neurons changed in control and GCaMP6 groups during licking behavior.

(A) Ca^{2+} signal of mice cholinergic neurons changed in the control group during licking behavior. Twelve responsive recordings were from five mice (B) Ca^{2+} signals of mice cholinergic neurons changed in the GCaMP6 group during licking behavior. Nine responsive recordings were from five mice. Y: trial number; X: time; colorbar unit, ΔF/F.

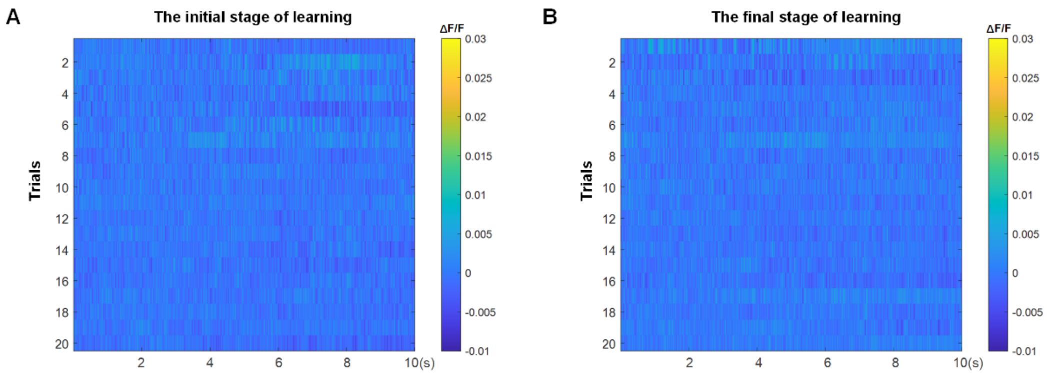

Supplementary Figure S4. Ca^{2+} signal of mice cholinergic neurons changed in control groups in the initial and final stage of go/no-go learning.

(A) Ca^{2+} signal of mice cholinergic neurons changed in control groups in the initial stage of go/no-go learning. (B) Ca^{2+} signal of mice cholinergic neurons changed in control groups in the final stage of go/no-go learning. Twenty responsive recordings were from five mice. Y: trial number; X: time; colorbar unit, ΔF/F.

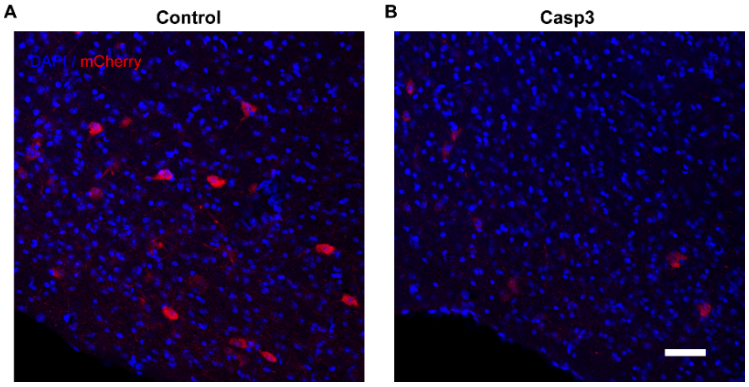

Supplementary Figure S5. Representative images of ChAT^{+} neurons in HMSc brain area separately from the control group and Casp3 group in the go/no-go tasks.

(A) mCherry signals of mice in control group. (B) mCherry signals of mice in Casp3 group.
